# Timing of menarche, childbearing and hysterectomy risk

**DOI:** 10.1016/j.maturitas.2008.09.025

**Published:** 2008-12-20

**Authors:** Rachel Cooper, Rebecca Hardy, Diana Kuh

**Affiliations:** MRC Unit for Lifelong Health and Ageing, London, UK

**Keywords:** Age at menarche, Parity, Age at first birth, Hysterectomy, Birth cohorts

## Abstract

**Objectives:**

To examine the associations of age at menarche, timing of first birth and parity with hysterectomy rates; to investigate whether these associations were independent of each other and other potential confounders and varied by reason for hysterectomy.

**Methods:**

Women from the Medical Research Council National Survey of Health and Development, a cohort followed prospectively since birth in March 1946 across England, Scotland and Wales, were studied. Survival analyses were used to assess the relationships between reproductive characteristics and hysterectomy.

**Results:**

Age at menarche was inversely associated with hysterectomy rates (adjusted Hazard Ratio for hysterectomy associated with a 1 year increase in menarche = 0.85 (95% CI: 0.77–0.95)). Parity was also associated with hysterectomy; women with ≥3 children experienced higher rates of hysterectomy than women with 1–2 children, who themselves experienced higher rates than nulliparous women. The associations of parity and age at menarche with hysterectomy were independent of each other and potential confounders. The association between age at menarche and hysterectomy was stronger for hysterectomies performed for fibroids than for other reasons.

**Conclusions:**

Our findings suggest that age at menarche is most likely acting to influence hysterectomy risk through its association with lifetime oestrogen exposure whereas parity is most likely acting through an effect on decision-making processes. This highlights the importance of considering both biological and social pathways to hysterectomy and confirms that medical need is not the only factor which needs to be considered when making treatment decisions for gynaecological problems.

## 1 Introduction

Existing evidence suggests that age at menarche, age at first birth and parity may all be associated with subsequent hysterectomy risk [1–18]. Most studies to examine either age at menarche or age at first birth have found inverse associations between these reproductive characteristics and hysterectomy with women who had an earlier age at menarche [2,7,9,10,19] or an earlier age at first birth [1,2,8,13,18–20] having increased risk of hysterectomy compared with women who experienced these events at later ages. The association between parity and hysterectomy is less consistent. While the majority of studies [2,4,5,8,12,13,15–17,19–21] have found a positive association between parity and hysterectomy, others have found no association [6,10,14,18,22] or a negative association [3].

There are a range of plausible pathways along which these three reproductive characteristics could influence risk of hysterectomy. For example, they could all act through their associations with lifetime exposure to oestrogen to influence the development of gynaecological conditions and hence medical need for hysterectomy. Parity could also be acting through an association with damage to gynaecological organs and the pelvic structure so increasing medical need for the procedure. Alternatively, these three characteristics could influence risk of hysterectomy through their effect on decision-making processes. Considerations women have to make when deciding whether to undergo hysterectomy such as desire to preserve fertility or conversely, desire to prevent risk of further pregnancies are associated with reproductive history. However, while existing studies suggest that patterns of childbearing and timing of menarche are associated with risk of hysterectomy, which of the plausible pathways underlie these associations has yet to be elucidated. It is also unclear whether all three factors are operating on the same or different pathways or whether at least one of the three reproductive characteristics is mediating or confounding the associations of the other reproductive characteristics with hysterectomy. Further, most existing studies have not examined the different reproductive characteristics together and have used self-reported retrospective measures of reproductive histories, which may introduce bias especially for age at menarche [23]. Other limitations of the majority of studies include a lack of control for potential confounding factors and a failure to examine variation in associations by reason for hysterectomy.

Using data from the Medical Research Council National Survey of Health and Development (NSHD) which has been prospectively collected across life, the objectives of this study were to examine associations of age at menarche, age at first birth and parity with subsequent hysterectomy risk, and to assess whether these associations were independent of each other and other potential confounding factors. We also aimed to examine variation in associations by reason for hysterectomy.

## 2 Methods

### 2.1 Study population

The NSHD is a socially stratified cohort of 5362 males and females followed up since their births across England, Scotland and Wales during 1 week in March 1946 [24]. Information on a wide range of health and social factors has been collected regularly with the most recent major data collections taking place in 1999 when the cohort were aged 53 years. In addition, a series of postal questionnaires focussing on women’s health were sent annually to the women in the cohort between ages 47 and 54 and at 57 years [25]. Of the 2547 females in the original cohort, by age 53 years 154 (6%) had died, 232 (9%) were living abroad, 296 (12%) had refused to participate and 87 (3%) were untraced.

### 2.2 Ascertainment of hysterectomy status

Women provided responses to questions asked at home visits in 1989 and 1999 (ages 43 and 53 years) and in the women’s health postal questionnaires about hysterectomy and oophorectomy and the timing of procedures. Hysterectomy was defined as any self-report of hysterectomy with or without oophorectomy and in the majority of analyses was grouped into two categories: no hysterectomy, including women who had reported an oophorectomy only; hysterectomy with or without oophorectomy. Women who had reported a hysterectomy but had an unknown date of procedure were excluded from analyses (*n* = 5).

Reasons for hysterectomy were ascertained from hospital records or where these were not available from responses to a self-completion questionnaire sent in 2005 to hysterectomised women (R. Cooper, unpublished PhD thesis, 2006) These data were used to create a reason for hysterectomy variable: no hysterectomy or oophorectomy; hysterectomy for: fibroids; menstrual disorders; prolapse; cancer; other known reason; unknown reason.

### 2.3 Measurement of reproductive characteristics

Age at menarche was recorded during medical examinations performed when cohort members were aged 14–15 years. At this time school doctors established whether the female members of the cohort had started their periods and if so, the month and year of onset. If a cohort member had not reached menarche by the time of the medical examination this was recorded. In the 1994 ‘Women’s Health in the Middle Years’ questionnaire, women were asked for the age in years at which their periods started. The information from these data collections was combined. Retrospective self-reports of age at menarche may be subject to recall bias – of the 946 women in the NSHD who had both measures of age at menarche available, only 43.6% self-reported the same age at menarche at age 48 years as had been recorded during the medical interviews at age 14–15 years (*κ* = 0.35) [23]. Therefore, information was taken from the medical interview at age 14–15 years if available with women’s report of age at menarche at age 48 years used only if they were known not to have reached menarche by age 14–15 years and the age they self-reported in 1994 was ≥14 years (*n* = 94). Two variables were created – a continuous variable and a categorical variable with four groups: ≤11; 12; 13; ≥14 years. The categorical variable has a slightly larger *N* as those women known not to have reached menarche by age 14–15 years could be included in the upper category even if they had not reported a valid age in 1994.

Information on live births was ascertained at data collections across adulthood. Age at first birth was considered as a continuous variable and also categorised into four groups: 15–20; 21–25; 26–30; ≥31 years. Women who were nulliparous were not included in analyses of age at first birth. As hysterectomy ends reproductive life if it is performed before menopause it can influence parity; to ensure parity occurs before hysterectomy in all women in analyses, parity achieved by age 30 years was used. This age was selected as most hysterectomies (i.e. 96%) occurred after this age, and it provided an accurate reflection of women’s parity at the end of reproductive life – most women in the NSHD had their children early in life (only 8% (*n* = 123) of parous women had their first child at an age > 30 years). Parity was considered as a continuous variable and also categorised into four groups (0; 1; 2; ≥3 children).

### 2.4 Potential confounding factors

Factors were identified *a priori* as potential confounders of the association between reproductive characteristics and hysterectomy. These were all factors which have previously been shown to be associated with hysterectomy [26–28] and also with reproductive factors [29–33].

Father’s occupational class was ascertained at age 11 years (with information taken from data collections at ages 15 or 4 years if missing at age 11 (*n* = 223)). This was classified according to the Registrar General’s social classification and categorised into four groups: I or II; III Non-manual; III Manual; IV or V. Educational level attained was recorded at age 26 years and categorised into five groups: Degree or higher; A levels or equivalent; O levels or equivalent; CSE, clerical course or equivalent; None. Body mass index (BMI) (kg/m^2^) was calculated using height and weight measurements self-reported at age 26 years and categorised as <20; 20–25; 25–30; >30 kg/m^2^.

### 2.5 Analysis

Cox’s regression models were used to test the associations between each of the three reproductive characteristics and subsequent hysterectomy rates. All models were run on the age time scale with follow-up in months. Assumptions of proportionality were assessed by examination of plots and testing for interaction between the reproductive characteristics and the age time scale. When examining both age at menarche and parity, follow-up was from the average age at menarche (12.6 years) until hysterectomy. In all models including age at first birth follow-up was from age 35 years until hysterectomy. This shorter length of follow-up was used for age at first birth because there was evidence of an interaction between age at first birth and time (*p* = 0.001) when follow-up from age 12.6 years was used. This interaction was found because, amongst a group of women all of whom are known to be parous, women cannot have had their hysterectomy before they have had their children. Follow-up thus needed to be started at a time when all women were ‘at risk’ of hysterectomy, i.e. after they had had their first child. Age 35 years was selected as 99.2% of women had had their first child by this age but most hysterectomies had not yet occurred. In all models follow-up times of women who had not had a hysterectomy were censored at the time when they last completed a questionnaire which assessed their hysterectomy status.

Based on a priori hypotheses that parity and age at first birth could be acting on the same pathway to influence hysterectomy, survival models were run in which only parous women were included and these two characteristics were adjusted for each other. In models including all women, the associations of age at menarche and parity with hysterectomy were then adjusted for each other. All models were then rerun with adjustments made for each potential confounder, and fully adjusted, i.e. with all potential confounders entered together.

A competing risks framework [34,35] was used to assess whether the association between each reproductive characteristic and subsequent hysterectomy rates differed by reason for hysterectomy. This involved running a separate set of Cox’s proportional hazards models for each category of reason for hysterectomy. For example, to test the associations between each of the three reproductive characteristics and hysterectomies for fibroids, women with hysterectomies for fibroids were coded as having a positive outcome whereas women with hysterectomies for any other reason were grouped with women who had not had a hysterectomy and their follow-up censored at the time of hysterectomy. This was repeated for each of the six categories of reason for hysterectomy.

## 3 Results

Of the 1797 women in the NSHD who had responded to at least one data collection since 1989, 403 (22.4%) had undergone a hysterectomy with or without oophorectomy by age 57 years. Of these women, 5 did not report a date of procedure and 2 did not provide any information on their hysterectomy status. The most common reason for hysterectomy was fibroids with 123 (30.5%) hysterectomies performed for this reason. 116 (28.8%) hysterectomies were performed for menstrual disorders, 38 (9.4%) for prolapse, 28 (7.0%) for cancer and 62 (15.4%) for other known reasons. Only 36 (8.9%) hysterectomies were performed for unknown reasons.

In unadjusted analyses, age at menarche was found to be inversely associated with hysterectomy rates (Table 1). Women who had an older age at menarche had lower rates of hysterectomy than women with younger ages at menarche. Tests of deviation from linearity were not significant suggesting that this association was linear on the log scale.

**Fig. 1:**
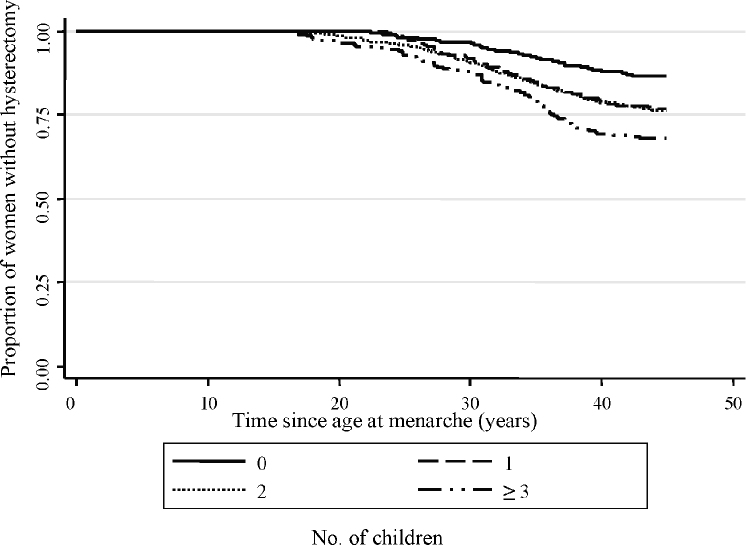
Kaplan–Meier survival estimates for hysterectomy by parity at age 30 years in the NSHD (*N* = 1746).

Parity was also found to be associated with hysterectomy – nulliparous women had the lowest rates of hysterectomy and women with ≥3 children had the highest. Fig. 1 clearly demonstrates that women with ≥3 children had the highest rates of hysterectomy at all ages while women with one or two children had similar hysterectomy rates to each other although these were still higher than the rate amongst nulliparous women. Tests of deviation from linearity were not significant suggesting that the positive association between number of children and hysterectomy rates was linear on the log scale. Women with later age at first birth had lower rates of hysterectomy than women with earlier age at first birth and this association was linear (Table 1).

When age at first birth and parity were included in the same survival model, the adjusted hazard ratios estimated were difficult to interpret because of collinearity. Age at first birth and parity were strongly associated – women who had an earlier age at first birth were more likely to have higher parity than women who had a later age at first birth (results not shown). Results from likelihood ratio tests comparing a model with both variables included to models with each variable entered individually suggested that there was no benefit in including both variables in the same model and therefore that these two reproductive characteristics act on the same pathway to influence hysterectomy risk. Due to the plausibility of the association between parity and hysterectomy it was concluded that age at first birth was associated with hysterectomy rates because it was a marker of parity.

When age at menarche and parity were adjusted for each other the point estimates of effect were not greatly altered (Table 2), suggesting that these two reproductive characteristics predict hysterectomy rates independently of each other. This would be expected given age at menarche and parity were not found to be associated with each other (results not shown). When additional adjustments were made for other potential confounders both associations were maintained.

On examining variation in associations by reason for hysterectomy, a significant inverse association between age at menarche and hysterectomy for fibroids was found – HR for hysterectomy for fibroids (95% CI) = 0.80 (0.68–0.90) per 1 year increase in age at menarche (*p* = 0.01). For each other group of reasons there were non-significant associations operating in the same direction with HRs ranging from 0.83 to 0.98. There were no clear differences in the pattern of association between parity and hysterectomy by reason for hysterectomy (results not shown).

## 4 Discussion

Age at menarche, age at first birth and parity were all found to be associated with hysterectomy in a nationally representative British birth cohort. Women with an earlier age at menarche, earlier age at first birth, and who were parous had higher rates of hysterectomy than women with later age at menarche, later age at first birth and who were nulliparous. The associations of age at menarche and parity with hysterectomy were maintained after mutual adjustment and adjustment for other potential confounders. The association between age at menarche and hysterectomy varied by reason for hysterectomy with a stronger association found when considering hysterectomies for fibroids than when considering hysterectomy for any other reason.

### 4.1 Comparison with other studies

The results from these analyses are consistent with the findings from the majority of existing studies. In those studies which examined the independence of the association between age at menarche and hysterectomy it was found, as in this study, that age at menarche was associated with hysterectomy independently of other factors [2,9]. The independence of the association between parity and hysterectomy varies between studies. Some have found, as in this study, that there is an effect of parity which is independent of other predictors [2,5]. However, in other studies parity was not independently associated with hysterectomy [9,11,20]. Differences in findings between studies could be explained by variations in the country of study, age of the study population or methods used.

Methodological problems in analysing the associations between hysterectomy and age at first birth and parity were identified in these analyses that had not been reported in other studies. These problems exist because both reproductive characteristics as well as influencing hysterectomy risk could themselves be influenced by hysterectomy. Women cannot have more children once they have had a hysterectomy. Further, if a woman is known to have had children she cannot have had a hysterectomy at any age before these births occurred. These problems were successfully overcome by using parity at age 30 years and running analyses of age at first birth with follow-up from age 35 years. That no other study had identified or attempted to deal with these methodological challenges is because so many existing studies were cross-sectional and had used logistic regression instead of survival analyses.

### 4.2 Explanation of findings

Age at menarche and parity were independently associated with hysterectomy suggesting that they operate on different pathways to influence hysterectomy risk.

Age at menarche is one of the main influences on the length of time across life during which a woman is exposed to oestrogen, with women who have an earlier age at menarche regularly exposed to oestrogen and other endogenous hormones from an earlier age and therefore for a longer duration by the time they reach middle-age. In addition, it has been found that women with earlier age at menarche may be exposed to higher concentrations of oestrogen across life than women with later age at menarche [36,37]. Higher levels of exposure to unopposed oestrogen are associated with the development of some major gynaecological conditions, most importantly fibroids, [38] one of the most common reasons for hysterectomy. That women in the NSHD who had earlier ages at menarche had higher rates of hysterectomy and, that this association was significant for hysterectomies for fibroids suggests that age at menarche influences hysterectomy risk through its association with oestrogen exposure.

Some other theoretically plausible pathways between age at menarche and hysterectomy can be discounted by the analysis findings. For example, the association between age at menarche and hysterectomy was not explained by other reproductive characteristics. Further, the inverse association between age at menarche and hysterectomy was maintained after adjustment for factors, including BMI and lifetime socioeconomic position which were identified as most likely to mediate or confound the association. This evidence along with the finding of a consistency of association across studies implicates a biological pathway and provides further support that oestrogen exposure is the link between age at menarche and hysterectomy. However, another potential biological explanation of this association is shared genetic risk factors. It is well established that age at menarche is influenced by genetic factors and has a high level of heritability [39,40]. The oestrogen receptor α gene has been identified as one gene which could influence age at menarche [41,42]. Some polymorphisms of this gene have also been identified as potential predictors of some gynaecological conditions including endometrial cancer [43] and endometriosis [44] and also hysterectomy [45]. It is therefore possible that age at menarche mediates the association between genetic factors and hysterectomy or that the association between age at menarche and hysterectomy is confounded by genetic factors.

Unlike age at menarche, parity acts in the opposite direction to that which would be expected if it was associated with hysterectomy via an association with oestrogen exposure. It has also been demonstrated that the association is not explained by parity’s association with BMI or lifetime socioeconomic position. There are however, some pathways between parity and hysterectomy which are plausible and could explain the association in the direction found. With each additional birth, the likelihood of damage to gynaecological organs and the pelvis having occurred and, the amount of damage suffered is likely to increase which may result in greater medical need for hysterectomy among women with higher parity. While this explanation cannot be fully discounted, this pathway would be unlikely to result in such similar associations between parity and hysterectomies for each different reason.

The lack of variation in association by reason for hysterectomy was found despite parity being protective against some gynaecological disorders including fibroids [38,46,47] and endometrial cancer [48], having no independent association with other disorders including menorrhagia [49] and a risk factor for others including prolapse [50]. This suggests that parity is more likely to be acting on social or decision-making pathways to influence risk of hysterectomy than on pathways which influence medical need for hysterectomy. The inconsistent findings between studies also suggest that parity is more likely to be acting on supply and demand factors rather than biological processes which influence hysterectomy risk. Women with greater numbers of children would be expected to be more likely than other women to request a hysterectomy to prevent further pregnancies, be less likely to decline the offer of a hysterectomy in order to preserve their fertility and may be more likely to be offered a hysterectomy by a doctor because of the reduced need perceived by the doctor to preserve fertility. This is supported by evidence from a study of women’s treatment preferences for menstrual problems in which nulliparous women found the idea of losing their fertility as a consequence of hysterectomy to be less acceptable than parous women [51]. This suggests that the loss of fertility, a consequence of hysterectomy for women who undergo the procedure pre-menopause, is an important consideration and influences women’s treatment choices and also possibly the doctor’s decision to offer a hysterectomy.

### 4.3 Strengths and limitations

By examining three reproductive characteristics together and, examining variation in associations by reason for hysterectomy it has been possible to identify the pathways to hysterectomy which are most likely to be operating. Only two studies [5,8] have previously examined variations in the associations between reproductive characteristics and hysterectomy by reason for hysterectomy and, neither of these examined age at menarche.

This study also has the important strength of being selected to be nationally representative. Although selective loss to follow-up could have introduced bias and reduced the generalisability of findings, comparisons of those women included in analyses with those women not included (results not shown) found no significant differences. Further, the availability of a range of prospectively collected measures from across life has allowed us to adjust for important potential confounders.

A potential limitation of our study is that we ascertained hysterectomy status using self-reports. However, use of self-reported hysterectomy is unlikely to have introduced bias as studies suggest that self-reports of hysterectomy and its timing are accurate [52,53] and it has been found that valid results are obtained from analyses whether self-reported or hospital recorded measures of hysterectomy are used [54].

### 4.4 Conclusions

We have found evidence of associations between age at menarche, parity and hysterectomy. These two reproductive characteristics appear to operate on different pathways with age at menarche most likely acting to influence hysterectomy risk through its association with lifetime oestrogen exposure and parity most likely acting through an effect on decision-making processes. These findings highlight the importance of considering both biological and social pathways to hysterectomy. They also clearly demonstrate that medical need is not the only factor which needs to be considered when making treatment decisions for gynaecological problems.

## Figures and Tables

**Table 1:** Unadjusted survival analyses of the associations between reproductive characteristics and hysterectomy in the NSHD.

Reproductive characteristic	*N* (%) [No. of hysterectomies]	Hysterectomy rate per 1000 women years (95% CI)	Hazard ratio for hysterectomy (95% CI)	*p*-Value
Age at menarche (years) (*N* = 1434)				
9–11	235 (16.39) [58]	6.14 (4.74–7.94)	1.00	0.01**
12	397 (27.68) [100]	6.23 (5.12–7.57)	1.01 (0.73–1.40)	
13	487 (33.96) [104]	5.25 (4.33–6.36)	0.84 (0.61–1.16)	
14 and above	315 (21.97) [52]	3.99 (3.04–5.23)	0.63 (0.43–0.92)	

Per 1 year increase^a^	1384 (100) [308]	5.46 (4.89–6.11)	0.88 (0.80–0.96)	0.005*

Parity at age 30 years (*N* = 1746)				
No. of children				
0	326 (18.67) [43]	3.13 (2.32–4.22)	1.00	<0.001**
1	339 (19.42) [76]	5.50 (4.39–6.89)	1.82 (1.25–2.64)	
2	741 (42.44) [168]	5.60 (4.82–6.52)	1.86 (1.33–2.60)	
≥3	340 (19.47) [105]	7.93 (6.55–9.60)	2.72 (1.91–3.88)	

Per 1 child increase^b^	1420 (100) [349]	6.12 (5.51–6.79)	1.22 (1.08–1.38)	0.001*

Age at first birth^b^ (years) (*N* = 1505) [follow-up from 35 years]				
15–20	358 (23.78) [89]	14.24 (11.57–17.53)	1.00	0.01**
21–25	647 (42.99) [144]	12.09 (10.27–14.24)	0.85 (0.65–1.11)	
26–30	378 (25.12) [78]	10.74 (8.60–13.40)	0.76 (0.56–1.02)	
≥31	123 (8.17) [20]	8.31 (5.36–12.88)	0.58 (0.36–0.95)	

Per 1 year increase	1505 (100) [331]	11.90 (10.68–13.25)	0.97 (0.94–0.99)	0.01*

a Total *N* less than *N* for analysis of age at menarche as a categorical variable as women with unknown age at menarche who were known not to have reached menarche by age 14 were included in the upper category but were necessarily excluded from analyses of age at menarche as a continuous variable.

b Nulliparous women excluded.

* *p*-Value from likelihood ratio test.

** *p*-Value from test for trend.

**Table 2:** Survival analyses of the associations between age at menarche, parity and hysterectomy in the NSHD (*N* = 1184).

Reproductive characteristic	Unadjusted hazard ratio for hysterectomy (95% CI)	Hazard ratio for hysterectomy (95% CI) adjusted for other reproductive characteristic	Fully adjusted^a^ hazard ratio for hysterectomy (95% CI)
Age at menarche			
Per 1 year increase	0.85 (0.77–0.94)	0.85 (0.77–0.94)	0.85 (0.77–0.95)

*p*-Value	0.002	0.001	0.003

Parity (No. of children)			
0	1.00	1.00	1.00
1	1.94 (1.23–3.06)	1.98 (1.25–3.13)	1.89 (1.20–3.00)
2	1.62 (1.07–2.46)	1.66 (1.10–2.52)	1.57 (1.03–2.40)
≥3	2.84 (1.84–4.39)	2.91 (1.88–4.48)	2.79 (1.80–4.34)

*p*-Value	<0.001	<0.001	<0.001

a Adjusted for other reproductive characteristic, father’s occupational class, educational level attained and body mass index.
